# Intussusception as a Presentation of Anisakis Infestation in the Global Era of Raw Fish Consumption: A Case Report and Literature Review

**DOI:** 10.7759/cureus.55232

**Published:** 2024-02-29

**Authors:** Satoshi Umemoto, Yasuhiko Fujita, Teruyoshi Amagai

**Affiliations:** 1 Surgery, Kagoshima Tokushukai General Hospital, Kagoshima, JPN; 2 Radiology, Kagoshima Tokushukai General Hospital, Kagoshima, JPN; 3 Faculty of Health Care Sciences, Department of Clinical Engineering, Jikei University of Health Care Sciences, Osaka, JPN

**Keywords:** dietary history, emergent general surgery, emergency medicine, intussusception, anisakidosis

## Abstract

Anisakiosis, also known as Anisakis larvae infestation, is an increasing parasitic infestation due to the worldwide spread of raw fish and shellfish consumption habits. We present a rare presentation of intestinal intussusception as a preoperative diagnosis and noticed it postoperatively due to Anisakis larvae. A 43-year-old man presented to the emergency department with abdominal pain around the umbilicus and vomiting for several hours. On physical examination at presentation, he had tenderness in the lower abdomen. His radiological studies showed a right-sided pseudo-kidney sign and ileo-colonic intussusception on ultrasound echography. His computed tomography images added findings of submucosal edema and wall thickening in the terminal ileum, swollen regional lymph nodes, and ascites. An urgent laparotomy was performed for ileo-colonic intussusception of an unknown cause. During the laparotomy, the ileocecal intussusception was manually reduced after dissecting the adhesion due to the previous appendectomy, and a partial ileotomy was undertaken using the Endo-GIA automatic anastomosis device. At the resected ileal wall surface, the presence of Anisakis larvae was noticed, and anisakidosis was diagnosed. The dietary history taken post-operatively revealed that he had eaten salmon, bonito, and squid sashimi four days prior to his emergency department visit. His postoperative course was uneventful, and he was discharged from the hospital on the fifth day postoperatively. Anisakiosis must be in the differential diagnosis of intussusception, and eating history seems like a cue to diagnose, and it might be meaningful to clinicians.

## Introduction

Anisakis larvae have a four-stage life cycle: eggs excreted by terminal hosts develop to stage 1 (L1), then hatch to free-swim (L2). L2 is ingested by crustaceans, and L2 matures to L3 in the first intermediate hosts. The final stage (L3) of Anisakis larvae remains in fish and/or squid as second intermediate hosts. These second hosts are consumed by humans, and L3-stage Anisakis larvae enter the human gastrointestinal (GI) tract as accidental infestations upon consumption of raw or inadequately processed L3-contaminated fish or squid [1]. The duration between the ingestion of infected fish and the onset of symptoms of Anisakis infestation depends on the anatomical sites in the GI tract where Anisakis larvae attach to the intestinal wall. In this case report, we would like to emphasize that anisakiosis might be overlooked in CT or ultrasonographic images that demonstrate intussusception. Anisakiosis must be included in the differential diagnosis of intussusception when considering radiologic images of intussusception, as anisakiosis is an increasingly parasitic infestation due to the worldwide spread of raw fish or shellfish consumption habits, the prevalence of which has quadrupled in the last decade [2]. A dietary history of taking raw or undercooked fish or shellfish during the last week might prevent overlooking anisakiosis in patients with an acute abdomen. We collected a case series of intussusception caused by anisakiosis and drew clinical specificities. Of them, 16 cases (76%) were reported from Japan. As Japanese foods have been spread worldwide, there is concern that the incidence of anisakiosis will increase in the future due to the global presence of raw fish in Japanese food; this report might be of great significance.

## Case presentation

**Figure 1:**
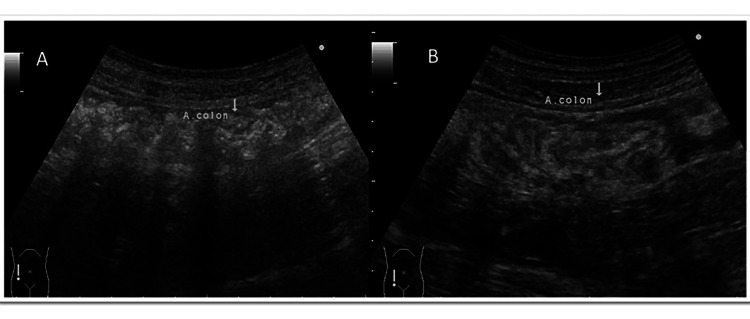
Abdominal ultrasound images of this case The abdominal ultrasound (US) images were taken in the right lower abdomen of the patient. (A) Intestinal edema was noted from the terminal ileum to the hepatic curvature of the transverse colon. Ascites was present around the ileocecal region. (B) Pseudo-kidney sign was observed.

**Figure 2:**
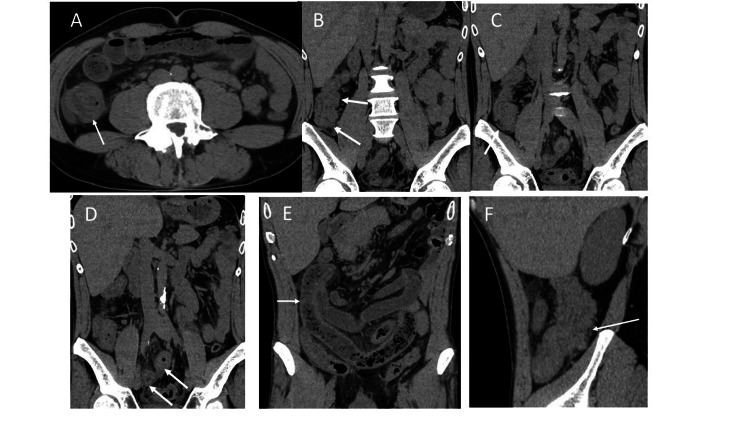
An abdominal computed tomography images of this case The abdominal CT images showed the intussusception of the ileum, and a dilated oral ileum was observed. (A, B) Arrows represent the intussusception image; (C) arrows represent the dilated ileum oral of the intussusception segment; (D) arrows represent the thickening ileal walls; (E) arrows represent the feces in the oral ileum of the intussusception segment; (F) arrows represent the incarcerated ileum in the ascending colon.

**Figure 3:**
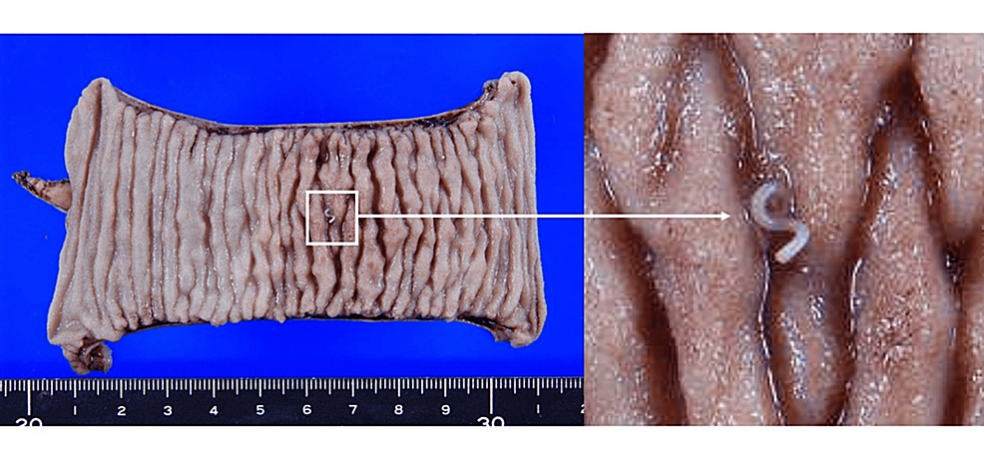
The resected ileum and Anisakis larva on the surface of the surgical specimen The Anisakis larva existed on the surface of the resected ileum. The photo on the right is an enlargement of a macroscopic photo of an Anisakis larva, whose head is anchored to the intestinal mucosa.

A 43-year-old man presented to the emergency department with abdominal pain around the umbilicus and vomiting for several hours. On physical examination, he had tenderness in the lower abdomen, no muscular defense, and weak rebound pain. His medical history includes an appendectomy at the age of 26. The physical and vital signs and laboratory findings at his first visit were as follows: height 174.2 cm, weight 67.8 kg, blood pressure 114/74 mmHg, heart rate 67 beats/min, temperature 36.9 °C, SpO2 96%, and laboratory data were white blood cell count 13,260 /μL (reference: 4,500-6,000), eosinophil 4.2% (reference: 1-9%), C-reactive protein 3.58 mg/dL, and the others were within normal ranges. His abdominal ultrasound echography showed intestinal edema from the terminal ileum to the transverse colon at the hepatic curvature and ascites around the ileocecal region. A right-sided pseudo-kidney sign was also identified, and ileo-colonic intussusception was diagnosed (Figure 1). The computed tomography (CT) images showed intussusception in the right lower abdomen, antegrade migration of the terminal ileum into the ascending colon, and a concentric stratified structure (Figure 2). In addition to submucosal edema and wall thickening in the terminal ileum, an increased concentration of adipose tissue was observed in the surrounding area; the dilated gastrointestinal tract with massive stool to the oral side of the intussusception area; the swollen regional lymph nodes; and ascites were observed. The examination did not reveal any findings suggesting malignancy. Under the diagnosis of ileo-colonic intussusception due to an unknown cause, a laparotomy was undertaken. The operative procedures were as follows: ileocecal intussusception was manually reduced after dissecting the adhesion due to previous appendectomy, and partial ileotomy with anastomosis was undertaken using the Endo-GIA automatic anastomosis device. Macroscopic pathological findings revealed an 11-cm length of the resected ileum, with a white linear Anisakis larva found on the surface of the specimen (Figure 3). Significant eosinophilic infiltration was observed histologically in the submucosal layer, mainly in the areas where the parasites were present. The postoperative dietary history revealed that he had eaten salmon, bonito, and squid sashimi four days prior to his emergency department visit. The postoperative course was uneventful, and he was discharged from the hospital on the fifth day postoperatively.

## Discussion

The nomenclature of Anisakis larvae infestations

The nomenclature expert group recommended four different categories: (1) “anisakidosis” which refers to the disease produced by *A. pegreffi*, *Contracaecum osculatum*, *Pseudoterranova azarazi*, *P. cattani*, *P. decipiens*, and *P. krabbei* [3]; (2) “anisakiosis” refers to the pathology caused specifically by the species Anisakis simplex s.s. [4]; and (3) “pseudoterranovosis” caused by the genus Pseudoterranova [5]; (4) “anisakiasis” is the general larval infestation, which is a parasitic disease caused by anisakid nematodes (worms) that can invade the stomach wall or intestine of humans [6]. However, in the literature, anisakiosis, anisakidosis, and anisakiasis have been confused and used interchangeably. In this article, anisakis infestations are collectively referred to as anisakiosis. When anisakidae larvae are ingested through the human GI tract, clinically, anisakiosis is classified into four categories as follows: gastric A, enteric A, ectopic A, in which larvae migrate into mesenteric blood streams to the liver and muscle, and an allergic reaction to anisakidosis.

The clinical profiles of enteric anisakiosis

The latest study, which analyzed 409 documents and 762 cases (of which 713 were case reports) through a half-century of literature review from 1965 to 2022, found that the dietary habit of eating raw fish such as sushi and sashimi spreads worldwide. There is an increasing need to include anisakiasis as a differential diagnosis in other countries. In terms of anatomical sites affected, the stomach and small intestine were the most common (35% and 24% of all reported cases, respectively), with the symptom of abdominal pain occurring within a few hours of ingestion of infected fish and within two to seven days in the case of gastric and intestinal anisakiasis, respectively [7]. To diagnose anisakiasis, after taking a key dietary history of ingesting raw or undercooked fish and shellfish, gastric anisakiosis is easily diagnosed and treated by endoscopy, but in cases where endoscopy is nearly impossible in the jejunum or ileum. In such cases, abdominal ultrasound or CT can be used to detect suspected intussusception, as in our case, followed by an emergency laparotomy for diagnosis and treatment. Especially in CT images, anisakid larvae show high intensity in CT with swelling of intestinal walls as allergic reactive findings and intussusception; these radiological-specific findings may lead to the correct diagnosis of anisakiasis.

Intussusception as the presentation of anisakiasis

The symptoms of anisakiasis are varied and include not only gastrointestinal symptoms but also respiratory symptoms, headaches, and central nervous system symptoms such as altered consciousness. Gastrointestinal symptoms include abdominal pain as well as symptoms caused by gastrointestinal obstruction [8]. Intussusception as a cause of obstruction is extremely rare, and as far as the literature has been reviewed, there have been 23 cases, including this one (Table 1) [3,5-7,9-22]. As mentioned above, the annual number of reported cases of anisakidosis in Japan between 2018 and 2019 was 19,737 [6]. The annual prevalence seems to be 10,000, and we collected 15 cases of intussusception caused by anisakiasis, including ours, in the past 35 years, as shown in the table. Calculating the prevalence from these, the prevalence of intussusception caused by anisakiasis infestation is 21/350,000 ≓ 6 of intussusception caused by anisakis infestation/100,000 of all cases with anisakis infestation. From this, we would conclude that intussusception caused by anisakis infestation seems to be extremely rare.

**Table 1:** Clinical profiles of adult cases with intussusception caused by anisakiasis From the literature, 21 cases of anisakiasis with intussusception as the chief complaint were collected, including this case. Abbreviations: m: male, f: female, ND: not defined.

No.	Year	Age	Sex	Symptoms	Treatment	Country	Ref
1	1989	61	m	Left lower abdominal pain	Partial jejunectomy	Japan	9
2	1990	38	m	Abdominal pain	Ileocecal resection	Japan	3
3	1994	66	m	Lower abdominal pain	Colonoscopy	Japan	5
4	1995	46	f	Abdominal pain, nausea	Partial jejunectomy	Japan	6
5	1998	35	f	Epigastralgia	Partial jejunectomy	Japan	10
6	2000	67	m	Abdominal pain	Barium enema	Japan	7
7	2000	69	f	Epigastralgia, melena	Colonoscopy and observation	Japan	11
8	2000	71	m	Abdominal pain, T-colon	Colectomy	Japan	12
9	2001	47	m	Abdominal pain	Ileocecal resection	Japan	13
10	2003	56	f	Epigastralgia, vomiting	Jejunectomy	Japan	14
11	2004	54	f	Epigastralgia, vomiting	Jejunectomy	Japan	15
12	2009	35	m	Epigastrium, vomiting	Jejunectomy	Japan	16
13	2010	41	m	Lower abdominal colicky pain, A-colon	Ileocecal resection	Japan	17
14	2013	67	f	Abdominal pain, trans colon	Colonoscopy	Japan	18
15	2014	52	f	Abdominal pain, vomiting	Right hemicolectomy	Italy	19
16	2017	48	f	Asc-trans colon	Right hemicolectomy	Spain	20
17	2019	59	m	Lower abdominal colicky pain	Colonoscopy	South Korea	21
18	2022	66	f	Terminal ileum	ND	Spain	22
19	2022	46	m	Right colon	ND	Spain	22
20	2022	55	m	Intestine	ND	Spain	22
21	2024	43	m	Abdominal pain, vomiting	Ileal resection with anastomosis, appendectomy	Japan	Present case

## Conclusions

We present a case of a 43-year-old male diagnosed with ileo-ileal intussusception caused by Anisakis infestation. The postoperative dietary history revealed that he had eaten sashimi fish four days prior to his emergency department visit. As the dietary habit of eating raw fish, such as sushi and sashimi, spreads worldwide, there is an increasing need to include anisakidosis as a differential diagnosis in patients with abrupt abdominal pain.
